# Characterization of the Bacteriophage vB_EfaS-271 Infecting *Enterococcus faecalis*

**DOI:** 10.3390/ijms21176345

**Published:** 2020-09-01

**Authors:** Gracja Topka-Bielecka, Sylwia Bloch, Bożena Nejman-Faleńczyk, Michał Grabski, Agata Jurczak-Kurek, Marcin Górniak, Aleksandra Dydecka, Agnieszka Necel, Grzegorz Węgrzyn, Alicja Węgrzyn

**Affiliations:** 1 Department of Molecular Biology, University of Gdansk, Wita Stwosza 59, 80-308 Gdansk, Poland; gracja.topka@phdstud.ug.edu.pl (G.T.-B.); bozena.nejman-falenczyk@ug.edu.pl (B.N.-F.); michal.grabski@phdstud.ug.edu.pl (M.G.); aleksandra.dydecka@phdstud.ug.edu.pl (A.D.); agnieszka.necel@phdstud.ug.edu.pl (A.N.); grzegorz.wegrzyn@biol.ug.edu.pl (G.W.); 2 Laboratory of Molecular Biology, Institute of Biochemistry and Biophysics, Polish Academy of Sciences, Kładki 24, 80-822 Gdansk, Poland; sylwia.bloch@ug.edu.pl; 3 Laboratory of Marine Biogeochemistry, Institute of Oceanology, Polish Academy of Sciences, Powstańców Warszawy 55, 81-712 Sopot, Poland; 4 Department of Molecular Evolution, University of Gdansk, Wita Stwosza 59, 80-308 Gdansk, Poland; agata.jurczak-kurek@ug.edu.pl (A.J.-K.); marcin.gorniak@ug.edu.pl (M.G.)

**Keywords:** bacteriophage, *Enterococcus faecalis*, genomic analysis, phage development, biofilm destruction, biodiversity of viruses

## Abstract

A newly isolated bacteriophage infecting *Enterococcus faecalis* strains has been characterized, including determination of its molecular features. This phage, named vB_EfaS-271, has been classified as a *Siphoviridae* member, according to electron microscopy characterization of the virions, composed of a 50 nm-diameter head and a long, flexible, noncontractable tail (219 × 12.5 nm). Analysis of the whole dsDNA genome of this phage showed that it consists of 40,197 bp and functional modules containing genes coding for proteins that are involved in DNA replication (including DNA polymerase/primase), morphogenesis, packaging and cell lysis. Mass spectrometry analysis allowed us to identify several phage-encoded proteins. vB_EfaS-271 reveals a relatively narrow host range, as it is able to infect only a few *E. faecalis* strains. On the other hand, it is a virulent phage (unable to lysogenize host cells), effectively and quickly destroying cultures of sensitive host bacteria, with a latent period as short as 8 min and burst size of approximately 70 phages per cell at 37 °C. This phage was also able to destroy biofilms formed by *E. faecalis*. These results contribute to our understanding of the biodiversity of bacteriophages, confirming the high variability among these viruses and indicating specific genetic and functional features of vB_EfaS-271.

## 1. Introduction

It is estimated that bacteriophages are the most abundant biological entities on Earth, as their number is estimated to be approximately 10^31^ virions [1,2]. They play a central role in the development of molecular biology and genetic engineering [2]. Bacteriophage λ, infecting *Escherichia coli* cells, may serve as an example of viruses that have been used as models in deciphering molecular mechanisms of basic biological processes, as well as to develop methods allowing genetic manipulations of a broad spectrum of applications [3]. On the other hand, the large number of existing bacteriophages implies their crucial ecological role; thus, characterization of phages that infect very different bacterial species is necessary to assess the effects of these viruses in the natural environment [4,5]. Moreover, phage therapy, i.e., the use of bacteriophages to treat bacterial infections, appears to be a new hope for the treatment of patients, especially in the era of the antibiotic resistance crisis [6]. Another medical aspect of phages is the phenomenon of phage-mediated bacterial virulence, as some phages code for toxins that determine the pathogenicity of various bacterial species [7]. Finally, although studies on phages have facilitated the development of sophisticated genetic engineering tools, bacteriophages are also dangerous factors that can destroy biotechnological production if the infecting bacteria synthesize desirable compounds in bioreactors [8,9,10].

In light of the abundance of bacteriophages and their unquestionable roles in the environment, science, medicine, and industry (particularly biotechnology), our knowledge of the biodiversity of these viruses is surprisingly scarce. The current number of complete genome sequences of bacteriophages is only slightly higher than 10,000 (see: US National Center for Biotechnology Information (NCBI). Available online: https://www.ncbi.nlm.nih.gov/genome/browse/#!/viruses/ (accessed on 27 August 2020)), which indicates that phage genomes are far underrepresented in genetic databases relative to their biological abundance. Even less complete knowledge is available on the biological characterization of bacteriophages, i.e., the characterization of their properties in host range, development inside host cells, sensitivity of virions to various conditions, and others. This problem has been underlined recently, and the enormous biodiversity of bacteriophages has been demonstrated even if a single habitat was considered [11]. Therefore, characterization of newly isolated bacteriophages, including determination of their molecular features, is necessary to expand the highly incomplete knowledge on these viruses.

Enterococci belong to natural microbiota in human gastrointestinal tract. However, it was found that following extensive antibiotic treatment of hospitalized patients, natural enterococci are eliminated causing reduction of thickness of the mucus layer which normally protects the gastrointestinal tract and outgrowth of hospital associated, multiantibiotic-resistant enterococcal strains [12,13,14,15,16]. There are two pathogenic species of enterococci causing major problems arising from nosocomial infections, *Enterococcus faecalis* and *Enterococcus faecium*; they are responsible for 85–90% and 5–10% enterococcal human infections, respectively [17,18]. These bacteria are considered as the third most common cause of nosocomial infections and primary sepsis [18,19,20,21]. Moreover, it appears that they cause about 25–50% deaths of hospitalized patients [22,23,24,25,26,27]. Apart from multiple antibiotic resistance of *E. faecalis* [18,28], another clinical problem is formation of biofilms by this bacterium, making infections particularly difficult to treat [29]. Therefore, there is an urgent need to develop alternative methods for treatment of *E. faecalis* infections. One of possibilities is the use of phage therapy, i.e., the use of bacteriophages to treat bacterial infections as these viruses can attack host cells, propagate inside them and eventually kill them liberating phage progeny [30]. However, since bacteriophages are specific not only to bacterial species but also strains, to develop effective phage therapy it is necessary to isolate and characterize a large collection of these viruses which reflect specificity to particular bacterial hosts [6]. Therefore, apart from gaining more complete knowledge about biodiversity of bacteriophages (mentioned in the preceding paragraph), isolation and characterization of newly discovered phages may have a practical aspect, facilitating development of phage therapy. Finally, phages infecting *E. faecalis* have been proposed as novel biotechnological tools [31,32], corroborating the importance of searching for previously unknown viruses which propagate on this bacterium.

Different bacteriophages infecting *E. faecalis* strains have been described. A comprehensive summary of characterization of known *E. faecalis* phages and their potential use in phage therapy has been published recently [33]. Generally, reports from recent years indicated a high biodiversity of bacteriophages specific for *E. faecalis* strains. Such reports can be exemplified by articles describing isolation and characterization of previously unknown phages of different properties [34,35,36,37,38,39,40,41,42,43,44,45], genetic modification of known phages and their use in experimental phage therapy (including effects on biofilms) [46,47], assessment of phages in therapy using animal models [48,49,50,51,52,53], and cloning of phage genes coding for specific lysins and characterization of the gene products in the light of killing *E. faecalis* cells [54,55,56,57,58,59,60].

In this work, we present the characterization of a newly isolated bacteriophage infecting *E. faecalis*. This characterization includes developmental features as well as molecular properties, including full genome sequence analysis, electron microscopy analyses of virions, and mass spectrometry analyses of phage proteins.

## 2. Results

The newly isolated bacteriophage vB_EfaS-271 was detected in a sample of urban sewage after testing for the presence of phages that may infect the *E. faecalis* 271 clinical strain (Supplementary Table S1). The isolation was performed by mixing the bacterial culture with filtered sewage accordingly to the protocol described in Section 4.2. Water samples were collected from Gdansk Wastewater Treatment Plant in Poland during the summer period (June, 2017). Below, we present the characterization of this bacteriophage.

### 2.1. Virion and Plaque Morphology

**Figure 1:**
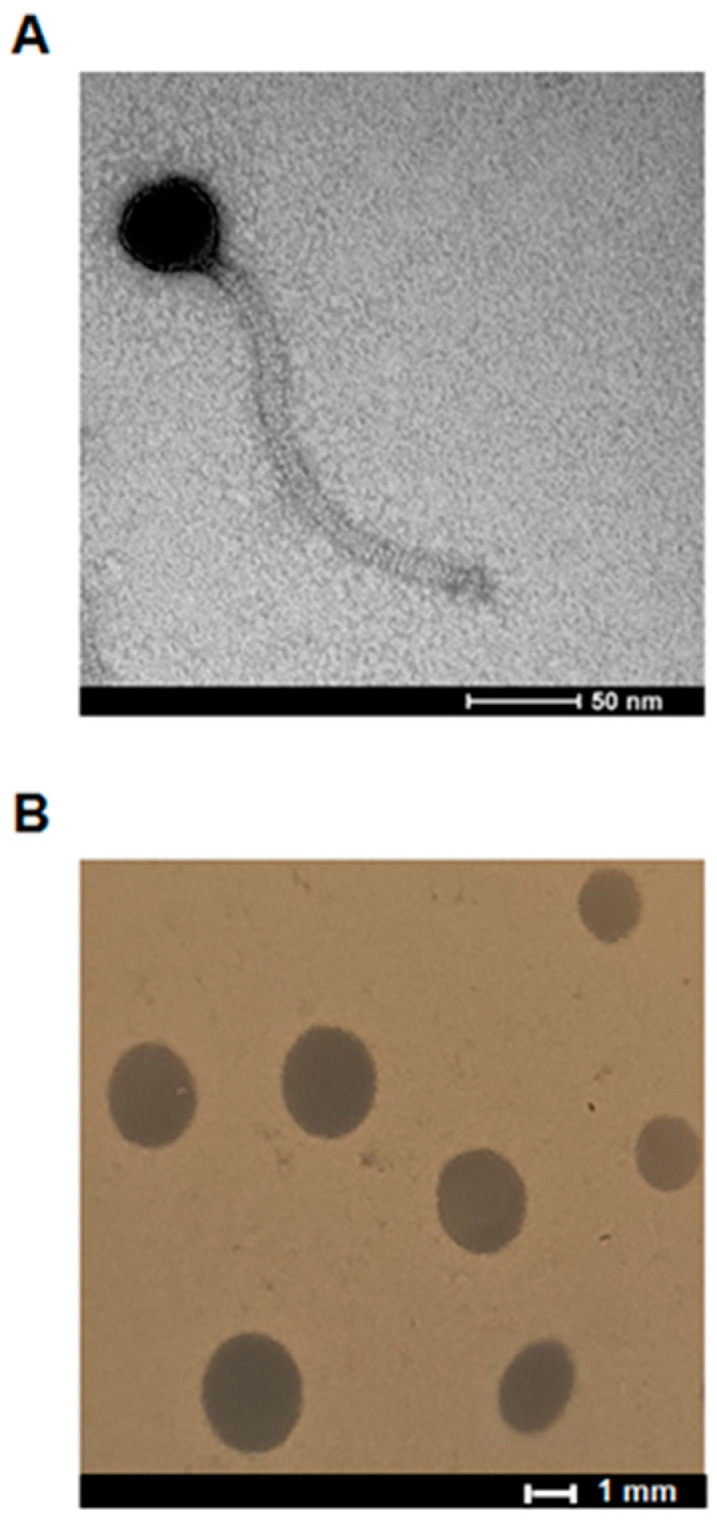
Morphologies of bacteriophage vB_EfaS-271 virion (**A**) and plaques formed by this bacteriophage on the lawn *E. faecalis* 271 strain using double-agar plates (**B**).

vB_EfaS-271 was propagated using the *E. faecalis* 271 host, and lysates were subjected to further analyses. Electron microscopy studies revealed that virions of this bacteriophage are composed of the head (diameter of 50 nm) and a long, flexible, noncontractable tail (dimensions: 219 × 12.5 nm) (Figure 1A). Such a morphology is characteristic of *Siphoviridae*; thus, vB_EfaS-271 has been classified to this group.

This phage formed clear plaques with a mean diameter of about 2 mm on the lawn of the *E. faecalis* 271 host (Figure 1B). Such a plaque morphology, together with analysis of the phage genome (Section 2.3) and inability to form prophages by vB_EfaS-271, indicate that this virus is a virulent bacteriophage.

### 2.2. Host Range

We tested the host range of phage vB_EfaS-271. Various strains belonging to Gram-positive and Gram-negative bacteria were tested as hosts (Table 1 and Supplementary Table S1). This phage did not infect any of the tested Gram-negative species: *Escherichia coli, Pseudomonas aeruginosa, Salmonella enterica* and *Shigella flexneri* as well as two of tested Gram-positive strains: *Enterococcus faecium* and *Staphylococcus sciuri* (Supplementary Table S2). However, vB_EfaS-271 was able to form clear zones on lawns of three strains of *E. faecalis*, clinical strains 271 and 272 and one commonly used V583 strain (Table 1). Therefore, we conclude that this phage is a narrow host range virus.

### 2.3. Phage Genome Analysis

**Figure 2:**
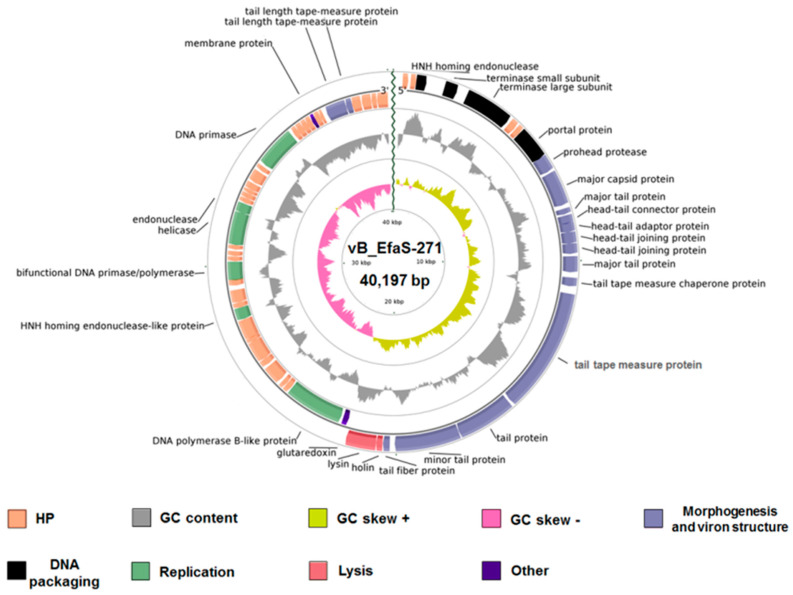
Circular map of the bacteriophage vB_EfaS-271 genome created using the BRIG platform. The innermost circle indicates GC content, where yellow and pink correspond to positive and negative GC skews, respectively. The gray ring shows the GC content. The ORFs with predicted functions are categorized into five groups: phage structure and morphogenesis (blue arrows), host lysis (red arrows), phage DNA replication (green arrows) and DNA packaging (black arrows) and others (dark blue arrows). For clarity, the hypothetical proteins (HP) are marked with orange arrows but not described on the map.

Bacteriophage vB_EfaS-271 DNA was isolated and subjected to nucleotide sequence determination (performed by the Genomed company, Warsaw, Poland). The 290,884 raw reads were obtained by using next generation sequencing technology and MiSeq Illumina platform. Assembly of the 99.48% of the raw data was accomplished using CLC Genomics Workbench and allowed to obtain the complete phage genome with an average coverage of 1527 times. The sequence of bacteriophage vB_EfaS-271 genome has been deposited in GenBank under accession number MT520979.1. The genome of vB_EfaS-271 consists of AT-rich linear double-stranded DNA of length 40,197 bp, with a G + C% content of 34% (Figure 2). The annotation information of this newly identified virus is summarized in Supplementary Table S3.

Bioinformatic analysis of vB_EfaS-271 DNA indicated that it contains 62 open reading frames (ORFs) with an average length of 586 bp, from which 23 ORFs are located on the direct strand of the virus genome and 39 ORFs were identified on the complementary strand. No tRNA genes were detected (Figure 2 and Supplementary Table S3). The majority of the ORFs (97%) possess methionine as a start codon, while vB_EfaS-271_09 and vB_EfaS-271_42 are initiated with GTG. The most frequently observed stop codon was TAA (38 ORFs; 61%), while TGA and TAG were identified in the sequences of 11 ORFs (18%) and 13 ORFs (21%), respectively. Furthermore, analysis of the vB_EfaS-271 genome also showed 23 putative promoters (score ≥ 0.9) and 16 putative Rho-independent terminators, among which 4 were defined as bidirectional putative terminators (score ≤ −4.0 kcal/mol) (Supplementary Table S4).

**Figure 3:**
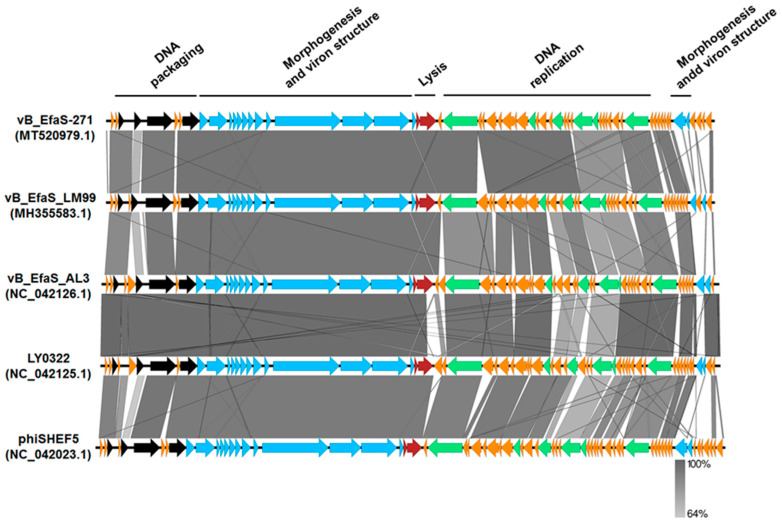
Multiple genome alignments of the vB_EfaS-271 bacteriophage and 4 other related enterococcal phages, vB_EfaS_LM99 (MH355583.1), vBEfaS_AL3 (NC_042126.1), LY0322 (NC_042125.1) and phiSHEF5 (NC_042023.1), created by using the EasyFig program. Open reading frames (ORFs) are shown as arrows to indicate the direction of transcription and are colored in accordance with their predicted functions: phage morphogenesis and virion structure (blue arrows), host lysis (red arrows), phage DNA replication (green arrows), and DNA packaging (black arrows). Orange arrows represent ORFs of unknown functions. The percentage of sequence similarity is shown as the intensity of the gray color. Vertical blocks between compared sequences indicate parts of the genome with at least 64% homology.

Interestingly, based on BLASTP analysis of the amino acid sequences of the vB_EfaS-271 proteins, putative functions could be attributed to 28 ORFs (45% of the ORFs), with the majority of them belonging to four modules: phage morphogenesis, host lysis, phage DNA packaging and phage DNA replication (Figure 3). Fifteen proteins were predicted to be involved in phage structure and morphogenesis, two in host cell lysis, four in phage DNA packaging, and six in virus DNA replication (Figure 3 and Supplementary Table S3). Moreover, the absence of integrases, repressors, transposases, recombinases, and excisionases supported the conclusion that vB_EfaS-271 is a lytic phage. Antimicrobial resistance genes and virulence factor homologs were also absent within the phage genome.

As indicated in Figure 3, the large subunit of terminase TelR of vB_Efas-271 phage is closely related to the large terminase subunits of other virulent *Siphoviridae* bacteriophages that infect *Enterococcus* hosts. Furthermore, based on BLASTN analysis, the nucleotide sequence of the genome of vB_EfaS-271 shares significant similarity to genomes of four enterococci viruses (coverage 89–90%, identity 92.64–97.28%): vB_EfaS_LM99 (MH355583.1) [41], vB_EfaS_AL3 (Nc_042126.1) [42], LY0322 (NC_042125.1) and phiSHEF5 (NC_042023.1) [43]. In addition, these five phages are highly similar in the organization of their genomes; however, the most significant differences occur in the region responsible for phage DNA replication (Figure 3). These differences may affect the rate of bacterial virus development and the efficiency of phage progeny formation.

**Figure 4:**
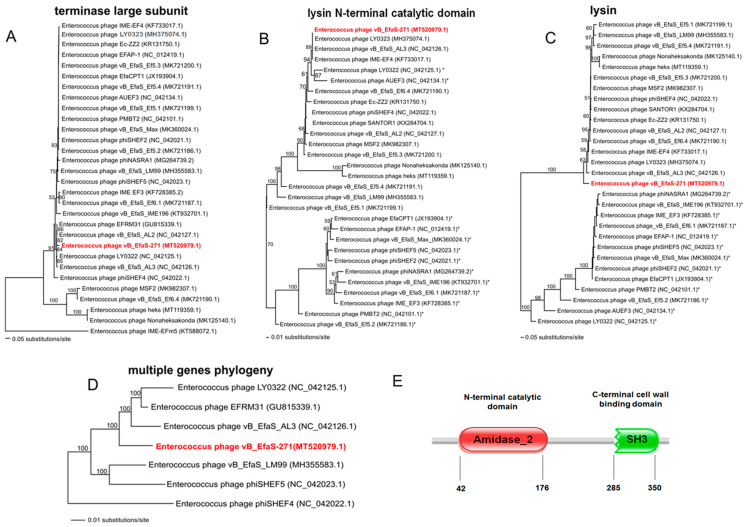
Neighbour-joining phylogenetic trees showing the phylogenetic position of vB_EfaS_271 phage (in red color) across other *Enterococcus* phages. Trees were constructed using PAUP * and based on: the sequence coding for terminase large subunit TerL (**A**), fragment of the lysin gene including sequence coding for an N-terminal catalytic domain of this protein (**B**) the complete sequence of the lysin gene (**C**), and sequences of 26 genes listed in the Supplementary Table S5 and coding for proteins of known functions (**D**). The reference sequences of the tested genes were collected from NCBI database. Bootstrap values > 50% calculated on the basis of 1000 resamplings are shown at the nodes. Branch lengths represented by a scale bar indicate the number of substitutions per site. Panel (**E**) shows domain organization of the lysin protein of the vB_EfaS_271 phage. The prediction was done with the Pfam database [61] (European Molecular Biology Laboratory, ELIXIR, Pram 33.1). Available online: http://pfam.xfam.org/ (accessed on 26 August 2020). Specific protein domains: N-acetylmuramoyl-L-alanine amidase (Amidase 2) and SH3 domain are marked with red and green colors, respectively. Numbers indicate positions of amino acids representing the beginning and end of the domain sequence.

To analyse phylogenetic relationships between investigated phage and other viruses, we have compared the nucleotide sequences of the gene coding for terminase large subunit of phage vB_EfaS-271 with the sequences of genes of the terminase large subunit of other phages. As shown in Figure 4A, the sequence of the terminase large subunit gene of bacteriophage vB_EfaS-271 indicates close relationship with *Enterococcus* phage EFRM31 (GU815339.1), belonging to the unclassified genus of the family *Siphoviridae* and *Enterococcus* phage vB_EfaS_AL2 (NC_042127.1) from *Efquatrovirus* genus. Interestingly, sequence similarity searches revealed that *Enterococcus* phage EFRM31 and bacteriophage vB_EfaS-271 show very low level of genome sequence coverage of ~34%. The sequence of the terminase large subunit gene of vB_EfaS-271 also shows a high level of identity with other *Enterococcus* phages such as LY0322 (NC_042125.1) and vB_EfaS_AL3 (NC_042126). Both of them belong to the genus *Efquatrovirus* of *Siphoviridae* and present high level of genome sequence identity with the studied phage (~97% identity).

We have also compared the nucleotide sequences of the lysin gene of vB_EfaS-271 with the sequences of other phages as lysins have significant therapeutic implications. The alignment of the DNA sequences revealed that the lysin genes of all analysed viruses share high nucleotide sequence homology only in a fragment of the gene (630 base pairs)—including sequence coding for an N-terminal catalytic domain (ECD). Interestingly, in the case of ECD, an *N*-acetylmuramoyl-l-alanine amidase domain (Amidase_2) was observed in all analyzed phages, including vB_EfaS-271 (Figure 4E). The phylogenetic tree based on this region is depicted in Figure 4B. The analysis showed that studied phage is most closely related to *Enterococcus* phages such as: vB_EfaS_AL3 (NC_042126.1), IME-EF4 (KF733017.1) and LY0323 (MH375074.1), belonging to the genus *Efquatrovirus* of *Siphoviridae*. We also present the tree of total lysin gene sequence for comparison (Figure 4C). The alignment of the rest of the gene was disrupted in 13 phages forming separate clade in the tree (Figure 4C). The disruptions in the sequences were caused by the presence of dissimilar domains in a C-terminal cell wall binding domain (CBD). Phages marked with an asterisks in Figure 4B,C have PET-M23 (ZoocinA) domain. Other phages, including vB_EfaS-271, have SH3 domain within the CBD region (Figure 4E). Interestingly, the phage ERMF31, closely related to vB_EfaS-271 as shown in Figure 4A, was not involved in this analysis because we did not find the homologous lysin gene in its genome. The phylogenetic position of the studied phage were changed when complete lysin gene sequences were used in the analysis (Figure 4C). vB_EfaS-271 is a sister phage to all other phages with SH3 domain in CBD.

Based on the tree of terminase large subunit and the results of genome sequence similarity searches, we selected phages for the multiple genes phylogeny. In this analysis, we compared a set of 26 genes coding for proteins of known functions in all phages but one—*Enterococcus* phage EFRM31 (GU815339.1), in which we had found only 14 genes of known functions homologous to the studied phage (Supplementary Table S5). Figure 4D presents the analysis of multiple genes coding for proteins of known functions in vB_EfaS-271 and selected viruses. To root the tree, we used the set of genes of *Enterococcus* phage phiSHEF4. The analysis confirmed the results obtained in the analysis of the terminase large subunit gene. The phages most closely related to the studied phage are: LY0322 (NC_042125.1), vB_EfaS_AL3 (NC_042126.1) and EFRM31 (GU815339.1).

### 2.4. Phage Protein Analysis

The purified lysate of vB_EfaS-271 obtained after propagation on the *E. faecalis* 271 host (sample withdrawn at time 1.5 h post-infection) was analyzed by mass spectrometry (MS). The names and short characteristics of all detected proteins are presented in Table 2. This identification analysis was based on fragmentation patterns of unique peptides and can also be verified in Supplementary Table S6.

We assigned 15 proteins of phage vB_EfaS-271 to annotated ORFs in the 7–156 kDA range. In this manner, 10 *in silico* predicted proteins related to virion structure and morphogenesis (vB_EfaS-271_9, vB_EfaS-271_10, vB_EfaS-271_11, vB_EfaS-271_14, vB_EfaS-271_15, vB_EfaS-271_16, vB_EfaS-271_18, vB_EfaS-271_19, vB_EfaS-271_20 and vB_EfaS-271_21) were confirmed. These structural proteins are the most highly-represented ones in the vB_EfaS-271 proteome in total mass spectrum counts (Table 2 and Supplementary Table S6). One of them is the major capsid protein (vB_EfaS-271_10) with molecular weight (MW) of 45.3 kDa and isoelectric point (pI) of 4.75 (Table 2 and Supplementary Table S6). It is worth to mention that amino acid sequence of this head protein is highly conserved among 17 enterococci phages deposited in GenBank with the coverage of 100% and identity of 85.9–97.1%. Moreover, homology searches revealed that the major capsid protein of the phage vB_EfaS-271 has the highest homology with 3 *E. faecalis* siphoviruses, named Ec-ZZ2 (coverage 100%, identity 97.1%; NC_031260.1) LY0323 (coverage 100%, identity 97.1%; NC_042125.1) and EFRM31 (coverage 100%, identity 96.9%; NC_015270.1). Interestingly, prohead protease (vB_EfaS-271_09) was also identified during MS analysis of the lysate of vB_EfaS-271 (Table 2 and Supplementary Table S6). This protein (MW = 21.1 kDa and pI = 5.36) belongs probably to the U35 protease prohead family and plays an essential role in phage capsid morphogenesis (Table 2 and Supplementary Table S3). Based on BLASTP analysis, the amino acid sequence of prohead protease of phage vB_EfaS-271 is the most similar to head maturation protease of bacteriophage EFAP-1 with the coverage and identity of 100%. Moreover, the presence of two head-tail joining proteins (vB_EfaS-271_14 and vB_EfaS-271_15) was also detected in the lysate containing vB_EfaS-271 virions. vB_EfaS-271_14 and vB_EfaS-271_15 proteins have MWs of 15.3 kDa and 14.2 kDa and pIs of 9.26 and 4.77, respectively (Table 2 and Supplementary Table S6). They are classified as a connector molecules that form the link between the elements of head and tail of the phage vB_EfaS-271 particle. BLASTP analysis revealed that both these proteins have the same high degree of similarity to head-tail joining proteins of the *Enterococcus* phage EFRM31 (NC_015270.1) with the coverage and identity of 100%. Among the detected structural proteins, there were also the fallowing phage-derived tail polypeptides: 2 major tail proteins (vB_EfaS-271_11 and vB_EfaS-271_16), tail tape measure protein (vB_EfaS-271_18), tail protein (vB_EfaS-271_19) minor tail protein (vB_EfaS-271_20) and tail fiber protein (vB_EfaS-271_21) (Table 2 and Supplementary Table S6). The vB_EfaS-271_11 (MW = 7.0 and pI = 6.61) and vB_EfaS-271_16 (MW = 19.9 and pI = 4.56) proteins are the main elements of the tail tube and represent the phage_TTP_1 superfamily (Supplementary Table S3). Protein sequence compression showed that the smaller major tail protein (vB_EfaS-271_11) has the amino acid sequence similar to the *Enterococcus* phage IME-EF4 (NC_023551.1) with coverage of 100% and identity of 93.9%, while the second tube protein (vB_EfaS-271_16) shares high level of similarity with phage tail-building protein of vB_EfaS_IME196 (NC_028990.1) with coverage and identity of 100%. Our MS analysis demonstrated also that the tail tape measure protein (vB_EfaS-271_18) has the MW of 156 kDA and pI of 9.5 (Table 2 and Supplementary Table S6). This tail length determination protein is responsible for the formation of the channel that is used for transmission of phage DNA into the bacterial host. Interestingly, the amino acid sequence of vB_EfaS-271_18 protein is almost identical to the tail tape measure proteins of 14 *Enterococcus* viruses with at least 85% homology (the best hit was obtained for phage vB_EfaS_LM99—coverage of 100% and identity of 99.3%). Moreover, vB_EfaS-271 structural proteins, named tail protein (MW = 78.9 kDa and pI = 5.43) and minor tail protein (MW = 88.4 and pI = 5.19), share above 96% amino acid sequence similarity with 4 enterococcal bacteriophages: vB_EfaS_LM99 (MH355583.1), vBEfaS_AL3 (NC_042126.1), LY0322 (NC_042125.1) and phiSHEF5 (NC_042023.1), suggesting that these viruses might infect the same host bacteria.

Prediction of the amino acid sequences, based on nucleotide sequences of genes of the investigated phage, indicated that the replication module of vB_EfaS-271 genome is represented by DNA primase (vB_EfaS-271_49) with MW of 60.7 kDa and DNA polymerase B-like protein (vB_EfaS-271_25) with MW of 87.5 kDa. DNA primase of vB_EfaS-271 is an acidic protein (pI = 5.81) and belongs to the group of RNA polymerases that synthesize oligoribonucleotide primers for DNA polymerase-mediated DNA replication (Table 2 and Supplementary Table S6). Its amino acid sequence is the most similar to the DNA primase of *Enterococcus* phage vB_EfaS_AL3 (coverage 100% and identity 99.6%). Interestingly, the vB_EfaS-271_49 protein is highly conserved among other 27 bacteriophages infecting *E. faecalis* strains with amino acid identity above 90.1%. DNA polymerase B-like protein of vB_EfaS271 virus is the member of POLBc superfamily with pI of 6.27 (Supplementary Table S3). The amino acid sequence of this enzyme is the most similar to DNA polymerase of bacteriophage vBEfaS_AL3 (NC_042126.1) with identity of 95.4%. Taking into account this bioinformatic analysis, it can be assumed that vB_EfaS271 and vBEfaS_AL3, two phages infecting *Enterococcus* strains, may reveal similar mechanism of DNA replication and the rate of development in host cells.

In addition, portal protein (vB_EfaS-271_08) was also identified (Table 2 and Supplementary Table S6). The vB_EfaS-271_08 protein is one of the main components of the DNA packaging machine with MW of 43 kDa and pI of 5.07. Interestingly, homology searches revealed that amino acid sequence of the portal protein of phage vB_EfaS-271 precisely corresponds to DNA packaging protein of 17 enterococcal viruses with identity above 90%.

Intriguingly, the presence of lysin (vB_EfaS-271_23), which is essential for host cell lysis, was detected in the sample containing vB_EfaS-271 lysate. The presence of a lytic protein in phage lysate might suggest either inclusion of the lysin in the virion (or its physical attachment to the capsid surface) or contamination of the lysate. If the latter option is true, this protein would have be extremely abundant to be able to contaminate the sample after several steps of purification of virions, including ultracentrifugation in the cesium chloride density gradient (see Section 4.16). Irrespective of the source of the lysin in the phage lysate, this protein can be classified as the element of the PGRP superfamily with MW of 39.9 kDa and pI of 8.34 (Table 2 and Supplementary Tables S3 and S6). It is worth to mention that BLASTP analysis showed the high level of identity between the vB_EfaS-271_23 protein and lysins produced by 15 other *Enterococcus* phages (100% coverage and ≥ 91.6% identity). The most similar lysin to vB_EfaS-271_23 protein is produced by *Enterococcus* phage IME-EF4 with homology of 99.7%.

Unexpectedly, only one vB_EfaS-271_28 (MW = 26.5 and pI = 4.78) gene product without similarity to a known virus protein could be classified as a protein of unknown function (Table 2 and Supplementary Table S6). However, its amino acid sequence is highly conserved among the group of 22 bacteriophages infecting *E. faecalis* strains (coverage 100% and identity > 90%). Taking into account this observation, one can speculate that this unknown protein may play an important role in the development of the phage vB_EfaS-271.

### 2.5. Sensitivity of Virions to External Conditions

We tested the sensitivity of vB_EfaS-271 virions to various external conditions, including various temperatures, pH conditions, solvents, detergents and disinfectants. The virions appeared relatively resistant to various temperatures and pH values, although they could not survive under extreme conditions (temperature of 95 °C and pH of 2). Sensitivity to organic solvents and detergents differed depending on the nature of the tested compound and were the lowest for cetyltrimethylammonium bromide (CTAB), ethanol and acetone (Table 3).

### 2.6. Bacteriophage Development

**Figure 5:**
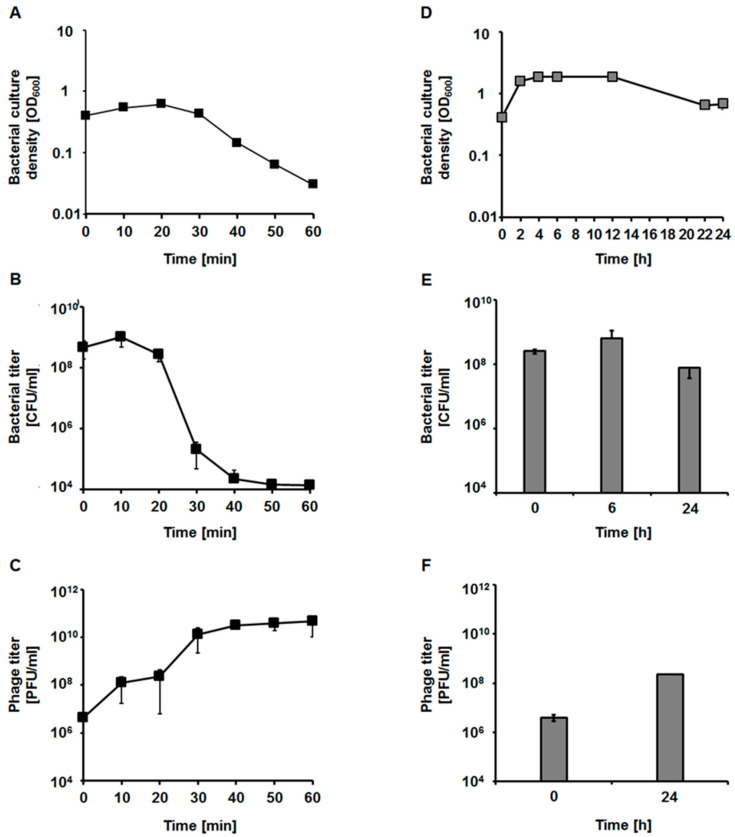
The lysis profile of host bacteria after infection of *E. faecalis* 271 (black squares and columns; Panels **A**–**C**) and 272 (gray squares and columns; Panels **D**–**F**) with the vB_EfaS-271 bacteriophage at an M.O.I. of 0.05. The results are presented as the bacterial culture density measured spectrophotometrically at OD_600_ (**A**), the number of bacterial cells surviving the phage infection per 1 mL (CFU/mL) (**B**) and the number of phages per 1 mL (PFU/mL) (**C**). The results are shown as the mean values ± SD from three biological experiments. Note that in some cases the error bars are smaller than sizes of symbols.

We determined parameters of phage vB_EfaS-271 development in the *E. faecalis* 271 and 272 host strains. When assessing the lysis profile in the 271 strain, we found that this phage causes lysis of sensitive bacteria relatively rapidly at 37 °C. Infection with multiplicity of infection (M.O.I.) of 0.05 caused the onset of bacterial culture lysis as soon as 20 min after the addition of phages (Figure 5A). Moreover, the number of colony forming units dropped significantly (Figure 5B) while number of plaque forming units (PFU) increased rapidly (Figure 5C). In contrast, development of vB_EfaS-271 in cells of the 272 strain was significantly slower and less efficient than in the 271 host, as indicated by the lysis of infected cultures only after overnight incubation and low phage titer (Figure 5D–F).

**Figure 6:**
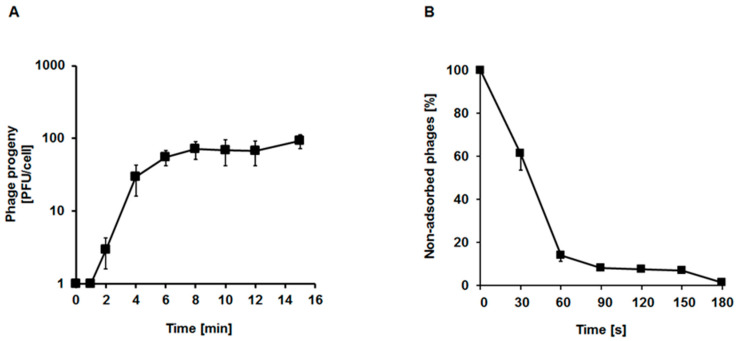
Kinetics of phage progeny production during one-step growth experiment (**A**) and the adsorption rate (**B**) of phage vB_EfaS-271 on the *E. faecalis* strain 271 measured at M.O.I. of 0.01 and 0.05, respectively. The results are shown as mean values ± SD from three biological experiments. Note that in some cases, the error bars are smaller than the sizes of the symbols.

One-step growth experiments were performed only after infection of the *E. faecalis* 271 host, as development of the phage in the 272 strain was too slow to allow for precise determination of the measured parameters (compare Figure 5D–F). These experiments confirmed that vB_EfaS-271 develops rapidly in the 271 host cells at 37 °C. The eclipse time was as short as 2 min, and the latency period was estimated to be 8 min. The average burst size was approximately 70 progeny viruses per cell (Figure 6A). Adsorption of vB_EfaS-271 on cells of the *E. faecalis* 271 host was also very efficient. Over 90% of the virions were effectively adsorbed within 90 s of incubation (Figure 6B).

### 2.7. Effects of the Bacteriophage on Bacterial Biofilm

**Figure 7:**
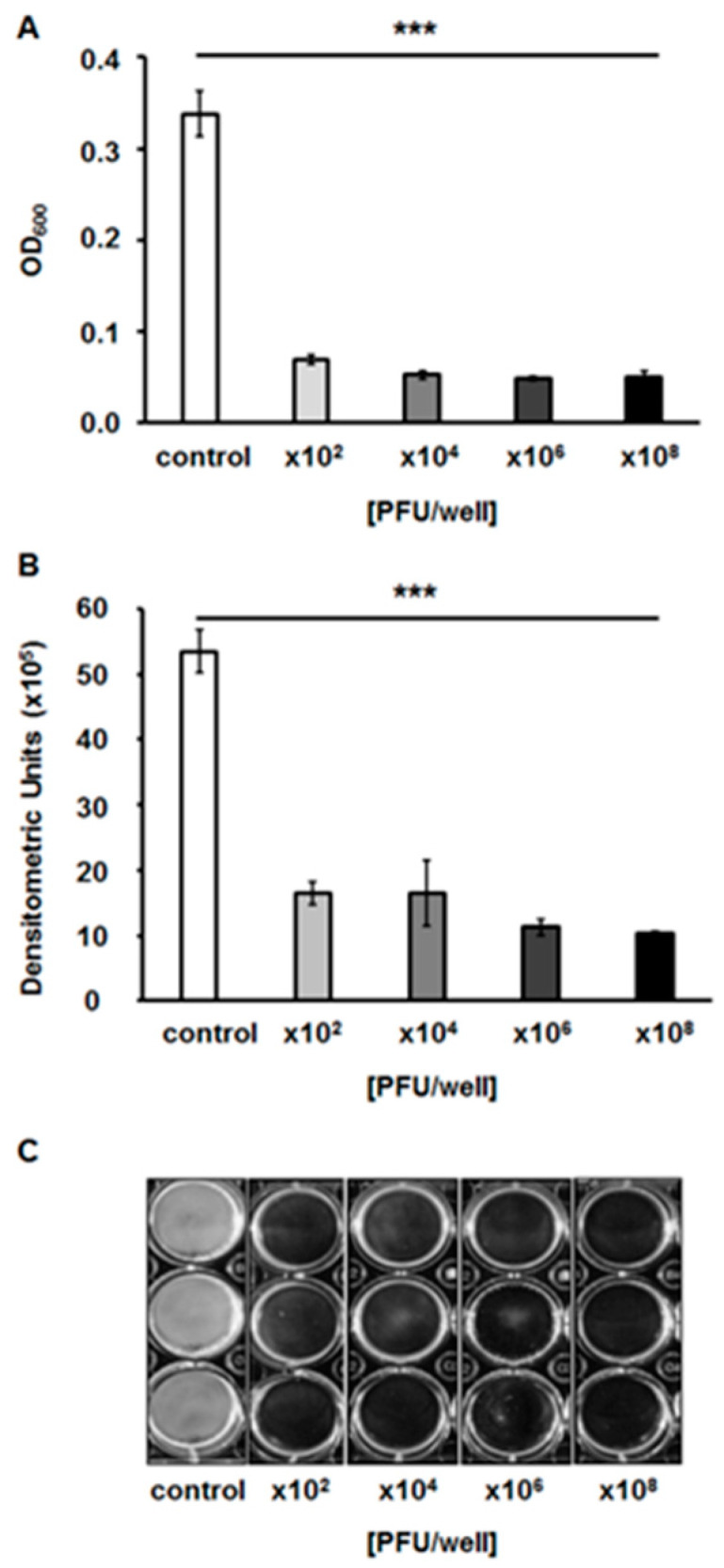
Bacterial biofilm density after a 24-h incubation with vB_EfaS-271 phage lysate added to final titers of 10^2^, 10^4^, 10^6^ or 10^8^ PFU/well. The results were estimated by measuring the optical density at a wavelength of 600 nm of the resuspended biofilms (**A**) or quantified densitometrically using the Quantity One program (**B**) from bacterial biofilm pictures (**C**). Error bars indicate the SD from triplicate experiments. Statistically significant differences (*p* < 0.001 in the *t*-test) between control and analyzed samples are marked by asterisks (***).

**Figure 8:**
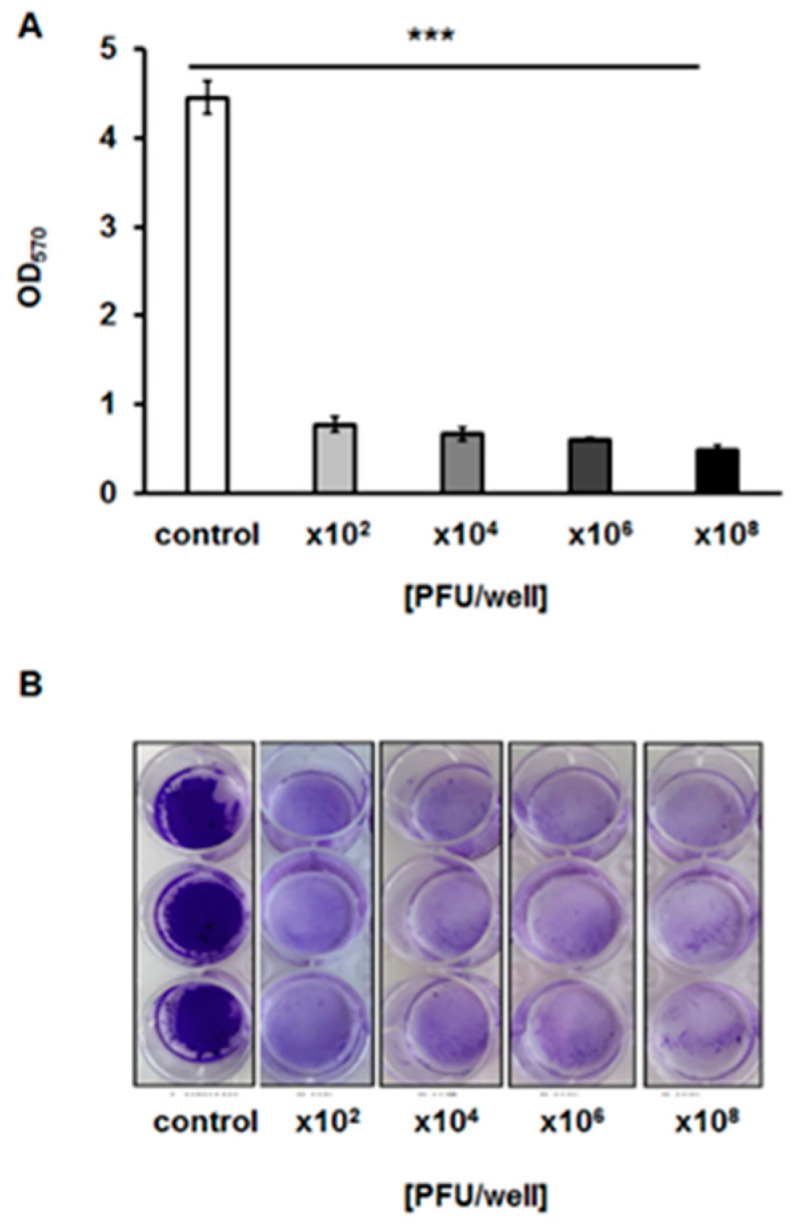
Biofilm biomass after a 24-h incubation with phage vB_EfaS-271 added to final titers of 10^2^, 10^4^, 10^6^ or 10^8^ PFU/well, as analyzed by the crystal violet staining method and shown as optical density values measured at a wavelength of 570 nm (**A**) or photographed (**B**). The results are presented as the mean values ± SD from three independent experiments. Statistically significant differences (*p* < 0.001 in the *t*-test) between control and analyzed samples are marked by asterisks (***).

**Figure 9:**
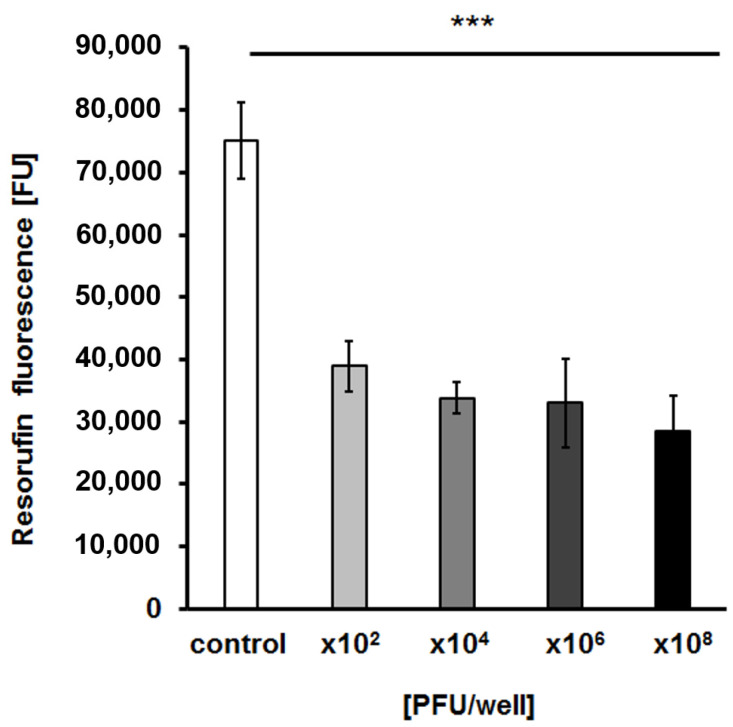
Metabolic activity/viability of bacterial cells in the biofilm after infection with phage vB_EfaS-271 estimated by the resazurin assay. Phage lysate was added to final titers of 10^2^, 10^4^, 10^6^ or 10^8^ PFU/well and incubated with the bacteria in the biofilm for 24 h. The results are presented as the mean values ± SD from three independent experiments. Statistical analyses were performed by *t*-test. Significant (*p* < 0.001) differences between the control without phage infection and particular variants of vB_EfaS-271 infection are marked by asterisks (***).

To test whether bacteriophage vB_EfaS-271 is able to destroy biofilms formed by *E. faecalis* 271, we analyzed biofilm density after treatment with the phage. We found that phage vB_EfaS-271 caused a significant decrease in the biofilm even if applied at the very low quantity of 10^2^ plaque forming units (Figure 7). Moreover, measurement of biofilm biomass with crystal violet staining indicated that this parameter dropped similarly to the biofilm density (Figure 8). The viability of bacterial cells in the biofilm was assessed by estimation of the metabolic activity in a test with resazurin. As in the biofilm density and biomass tests, the viability of *E. faecalis* 271 in the biofilm decreased significantly in the presence of phage vB_EfaS-271, even at the lowest tested dose of the virus (10^2^/well) (Figure 9). Therefore, we conclude that phage vB_EfaS-271 is effective in destroying the biofilm formed by *E. faecalis* 271.

## 3. Discussion

Bacteriophage vB_EfaS-271, infecting *E. faecalis* cells, was isolated from urban sewage, and its characteristics, including molecular properties, are presented in this report. Since bacteriophages are the most abundant biological entities on Earth and play crucial roles in the environment, science, medicine and industry, characterization of newly isolated phages is important, particularly in light of our incomplete knowledge about these viruses [1,2,3,4,5,6,7,8,9,10,11].

The investigated bacteriophage revealed a narrow host range, as it is able to infect only a small subset of *E. faecalis* strains. On the other hand, it adsorbs on the host cells very efficiently (over 90% of phages were effectively adsorbed after 90 s of incubation) and develops rapidly (with a latent period of 8 min) in the sensitive bacterial cells, giving an average burst size of approximately 70 phages per cell. Interestingly, it is effective in destroying bacterial biofilms formed by *E. faecalis* 271. Analysis of the genome sequence of vB_EfaS-271 indicated its similarity to some other phages infecting enterococci, including the modular character of the distribution of genes. The major modules contain genes coding for proteins involved in DNA replication, morphogenesis, packaging and cell lysis.

We suggest that the DNA replication module of the vB_EfaS-271 genome may be of particular interest. This module differs considerably from analogous regions of enterococcal phages. It consists of genes coding for putative DNA polymerase B-like protein, bifunctional DNA primase/polymerase, two endonucleases, helicase and primase. Such an expanded set of phage-encoded replication-related enzymes is relatively rare in bacteriophages with genomes that are approximately 40 kb in size. Therefore, one might speculate that well-developed specific replication machinery might have evolved to ensure particularly efficient phage genome replication. Such a hypothesis may be corroborated by the observation of rapid lytic development of phage vB_EfaS-271 reported in this work. This might allow efficient production of phage progeny under various environmental conditions, as well as effective infection of host cells included in biofilms. In fact, vB_EfaS-271 has been demonstrated to be effective in destroying *E. faecalis* 271 biofilm even at very low virion doses. Therefore, we suggest that further molecular characterization of replication enzymes may be of special interest and can lead to discoveries of novel properties of these proteins, especially since our knowledge of the variability and diversity of phage-encoded DNA polymerases is relatively poor. In fact, recent reports on the characterization of other enterococcal phages indicated that this group of viruses may have interesting and unique features, pointing to their high biodiversity [39,41,42,43,44,45,62,63,64,65].

Apart from genes coding for the expanded replication machinery, allowing for efficient lytic development of the phage, the vB_EfaS-271 genome contains no genes encoding proteins potentially involved in lysogenization. A lack of formation of prophages has been confirmed experimentally. Therefore, this phage in strictly virulent, replicating and developing rapidly in infected bacterial susceptible host—*E. faecalis* 271. One the other hand, development of vB_EfaS-271 was very slow and of low efficiency in another host strain, *E. faecalis* 272, indicating a high specificity of this bacteriophage. Narrow host range has also been confirmed by studies on several *E. faecalis* strains. The presence of the phage-encoded lysin protein in a highly purified bacteriophage lysate, detected by MS, was an intriguing discovery. This might suggest either the presence of the lysin in the virion (or attached to the phage capsid surface) or contamination of the lysate (which would be possible only if this protein is extremely abundant during the last step of bacteriophage development). Irrespective of which scenario is true, these results suggest very efficient mechanism of the cell lysis caused by vB_EfaS-271.

Finally, one might ask what can be practical applications of vB_EfaS-271? Since this phage is highly virulent, its genome lacks any identified genes coding for toxins or other proteins potentially deleterious to humans, and it is effective in destroying biofilms formed by *E. faecalis*, it is tempting to propose that it might be effectively used in phage therapy. However, vB_EfaS-271 is a narrow host range phage, able to infect only a relative small group of *E. faecalis* strains (including clinical isolates). Thus, its application in phage therapy may be limited. On the other hand, the lysin encoded in the vB_EfaS-271 genome appears to be particularly effective, suggesting its potential applications as antibacterial agent. There are also other interesting proteins encoded by this phage which might be considered as tools in biotechnological application. The examples are DNA polymerase and endonucleases, especially as DNA replication of this phage appears to be very effective, suggesting favorable properties of these enzymes in their putative use in genetic engineering.

## 4. Materials and Methods

### 4.1. Bacterial Strains and Growth Conditions

The bacterial strains used in this study are listed in Supplementary Table S1*. E. faecalis* isolates come from urine samples from patients of Specialist Hospital of St. Wojciech in Gdańsk (Poland) [66] or urban sewage samples from Department of Water and Waste-Water Technology of Gdansk University of Technology [67,68]. Commonly used *E. faecalis* strains OG1RF and V581 were obtained from National Medicines Institute (Poland) [69,70,71,72]. The rest of tested strains were from the collection of the Department of Molecular Biology of the University of Gdansk (Poland) [73,74,75,76,77]. All liquid bacterial cultures were grown with aeration at 37 °C in a shaking incubator (200 rpm). The plates with solid medium were incubated at 37 °C for 24 h. *Enterococcus* sp. and *Shigella flexneri* were cultured in Tryptic Soy Broth (TSB; BTL Company, Shiplay, UK) or plated onto solid Tryptic Soy Agar (TSA, BTL Company) supplemented with 1% glucose (Polish Chemical Reagents, Gliwice, Poland). For all phage experiments, *E. coli*, *Pseudomonas* sp., *Salmonella enterica* and *Staphylococcus* sp. were grown in Luria-Bertani liquid medium (LB; Lab Empire) or on LB solid medium with 1.5% bacteriological agar (BTL Company). The *E. faecalis* 271 strain was used for biofilm formation in flat-bottomed 12-well plates. Biofilms were grown in TSB medium with 0.5% glucose at 37 °C for 24 h.

### 4.2. Isolation of the vB_EfaS-271 Bacteriophage from Urban Sewage

vB_EfaS-271 was isolated from urban sewage (Gdansk Wastewater Treatment Plant, Poland) according to the procedure described earlier [78], with some modifications. The clinical *E. faecalis* 271 strain was used as a host strain for phage isolation. The sewage sample was centrifuged at 10,000×  *g* for  10 min at 4 °C, and the supernatant was filtered twice by using sterile membranes with pore sizes of 0.45 μm and 0.22 μm (Sigma-Aldrich, St. Louis, MO, USA) to remove bacterial debris. An exponentially growing bacterial culture of *E. faecalis* 271 (3 × 10^8^ CFU/mL) was mixed with 5 mL of filtered sewage and incubated with shaking at 37 °C for 24 h. After overnight incubation, the sample was centrifuged at 10,000× *g* for  10 min at 4 °C, and the collected supernatant was refiltered by using sterile membrane with pore sizes of 0.22 μm (Sigma-Aldrich). Phages were subsequently detected by the conventional double-layer agar method described elsewhere [11]. Briefly, 1 mL of overnight culture of *E. faecalis* 271 was mixed with 0.1 mL of phage solution. The mixture was added to 2 mL of soft TSB agar supplemented with 0.4% agarose (Sigma Aldrich) and then quickly poured onto the TSA plate. After overnight incubation at 37 °C, the single plaques were transferred to separate flasks with a mid-log phase culture of *E. faecalis* 271 and cultured at 37 °C until lysis occurred. After chloroform extraction, the phage lysate was 10-fold diluted in TM buffer (10 mM Tris-HCl, 10 mM MgSO_4_, pH 7.2) and replated on a lawn of host bacteria. The procedure of single plaque isolation was repeated three times.

### 4.3. Preparation of the vB_EfaS-271 Lysate

The *E. faecalis* 271 host was grown in TSB medium at 37 °C to an OD_600_ of 0.3. Phage vB_EfaS-271 lysate was added to the bacterial culture at an M.O.I. of 0.1 and subsequently incubated at 37 °C for 1.5 h with shaking. After lysis of the cells, the obtained phage lysate was treated with 4% chloroform for 15 min. In the next step, host cell debris was removed by centrifugation at 4000× *g* for 10 min at 4 °C. The supernatant was collected and diluted 10-fold in TM buffer (10 mM Tris-HCl, 10 mM MgSO_4_; pH 7.2). Then, a volume of 2.5 μL of each dilution of phage lysate were spotted onto the double agar layer. After overnight incubation at 37 °C, the number of plaques was counted. The phage titer was determined as plaque-forming units per ml (PFU/mL). The phage stock was kept at 4 °C until use.

### 4.4. Determination of the Bacteriophage vB_EfaS-271 Host Range

The host range of bacteriophage vB_EfaS-271 was determined by a standard spot test as described earlier [11]. The bacterial strains used in this experiment are presented in Table 2. Briefly, 1 mL of overnight bacterial culture was mixed with 2 mL of soft agar (LB with 0.7% agar or TSB with 0.4% agarose) and poured onto plates filled with bottom agar (LB with 1.5% agar or TSA). The phage lysate of vB_EfaS-271 was diluted in TM buffer (10 mM Tris-HCl, 10 mM MgSO_4_; pH 7.2), and 3 μL of each dilution was spotted onto the top agar. After a 24-h incubation at 37 °C, bacterial sensitivity to vB_EfaS-271 infection was confirmed by the presence of clear plaques at the sites of virus application. The obtained results were differentiated into two groups: clear zone (+) or no plaques (−).

### 4.5. Electron Microscopic Examination of Phage vB_EfaS-271 Morphology

Transmission electron microscopy (TEM) analysis of the morphology of vB_EfaS-271 virions was performed in the Laboratory of Electron Microscopy, Faculty of Biology, University of Gdansk, Gdansk, Poland. The lysate of vB_EfaS-271 was concentrated in the presence of 10% polyethylene glycol 8000 (PEG8000; Lab Empire, Rzeszów, Poland) overnight at 4 °C. The precipitated virions were pelleted by centrifugation at 8000× *g* for 20 min at 4 °C and resuspended in TM buffer (10 mM Tris-HCl, 10 mM MgSO_4_; pH 7.2). After extraction with chloroform, the purification of vB_EfaS-271 virions was carried out by using the cesium chloride density gradient centrifugation method described previously [79]. Phage particles were negatively stained with 3% uranyl acetate (pH 4.5) for 15 s and then observed under a Philips CM 1000 electron microscope (Philips, Eindhoven, The Netherlands).

### 4.6. Examination of the Morphology of vB_EfaS-271 Plaques

The morphology and size of the vB_EfaS-271 plaques were determined by using the double-layer agar method. The phage lysate was serially diluted in TM buffer (10 mM Tris-HCl, 10 mM MgSO_4_, pH 7.2). Then, 0.01 mL of each dilution was mixed with 1 mL of *E. faecalis* 271 overnight culture and 2 mL of TSB soft agar supplemented with 0.4% agarose. The mixture was quickly poured onto a plate filled with bottom TSA agar. After overnight incubation of the plates at 37 °C, the plaque morphology and diameter were determined.

### 4.7. The Effects of External Factors on the Stability of vB_EfaS-271 Virions

The sensitivity of vB_EfaS-271 virions to various physical and chemical factors, such as temperature, pH, detergents, organic solvents and osmotic shock, was tested according to the protocols described earlier [11]. The incubation times of lysates with individual factors are listed in the Table 3. Additionally, the stability of the phage particles was tested against five disinfectants used in the laboratory [80]. Each analysis was repeated three times.

### 4.8. vB_EfaS-271 Phage Adsorption Rate Assay

Overnight culture of the host strain was 100-fold diluted in TSB medium and incubated with shaking at 37 °C to an OD_600_ of 0.4. Three samples of 1 mL each were centrifuged at 2000× *g* for 10 min at 4 °C, and the obtained pellets were washed with cold 0.85% sodium chloride. The rinsing procedure was repeated three times, and then the bacterial pellets were suspended in 0.5 mL of TSB medium. After a 15-min incubation at 37 °C, the phage lysate was added to each tube at an M.O.I. of 0.05. Samples were collected every 30 s and centrifuged at 6000× *g* for 1 min at room temperature (RT). The pellets were discarded, and the supernatants were diluted in TM buffer (10 mM Tris-HCl, 10 mM MgSO_4_; pH 7.2). Titration of the non-adsorbed phages was performed onto the host lawn. The samples harvested at time 0 were considered 100% of un-adsorbed phages. Other values were calculated relative to this score. The obtained results are presented as the mean values ± SD from three independent experiments.

### 4.9. One-Step Growth Experiment

A one-step growth experiment was performed according to a procedure described earlier [81], with some modifications. Briefly, the *E. faecalis* 271 host was grown to an OD_600_ of 0.4 at 37 °C. Then, 10 mL of bacterial suspension was centrifuged at 4000× *g* for 10 min at 4 °C. The obtained pellet was suspended in 1 mL of fresh TSB medium supplemented with 3 mM sodium azide (Sigma-Aldrich) and then mixed with phage lysate at an M.O.I. of 0.01. Following a 10-min incubation at 37 °C, the non-adsorbed phage particles were removed by centrifugation at 4000× *g* for 10 min at 4 °C. The washing step was repeated three times. The bacterial pellet was resuspended in 1 mL of TSB medium with 3 mM sodium azide, and then 0.025 mL of the mixture was transferred into 25 mL of fresh TSB medium (time 0). The infected bacterial culture was aerated in an incubator shaker at 37 °C for 15 min. The number of infective centers was estimated from samples taken 1 min after infection by plating under permissive conditions. Due to the fact that at M.O.I. 0.05 (used in other experiments) infection centers were not counted, the M.O.I. value has been decreased to 0.01 in this case. Both M.O.I. values are very low and recommended in this type of analysis. Thanks to this, the experiment procedure did not have to be modified. Samples were withdrawn at the indicated times, and the phages were titrated. Burst size was calculated as the ratio of phages released at the appropriate time to the initial number of infective centers. The obtained results are presented as the mean values ± SD from three independent experiments.

### 4.10. Lysis Profile of the Bacterial Hosts after vB_EfaS-271 Infection

The lysis kinetics of infected cells and the number of phage progeny released during the lytic development of vB_EfaS-271 in the hosts were determined according to a protocol described earlier [82], with some modifications. Overnight cultures of bacterial strains were 100-fold diluted in TSB medium and incubated with shaking at 37 °C to an OD_600_ of 0.4. The phage stock solution of vB_EfaS-271 was added to 4 × 10^8^ *E. faecalis* 271 and 272 cells to an M.O.I. of 0.05. Bacterial growth after phage infection was monitored by measuring the OD_600_. During this experiment, the number of bacterial cells per ml (CFU/mL) and the number of phage particles per ml (PFU/mL) were also examined. To estimate the CFU/mL, 0.1 mL of bacterial sample was collected at the indicated times and then 10-fold diluted in 0.85% sodium chloride. In the next step, 0.04 mL of the appropriate dilution was spread onto a TSA agar plate. After overnight incubation at 37 °C, the bacterial survivors were counted, and the CFU/mL was determined. To calculate the phage titer, the samples were harvested at each time point and centrifuged at 2000× *g* for 5 min at room temperature, and then the supernatant was titrated onto the host lawn. Following overnight incubation at 37 °C, the phage titer was determined. This experiment was repeated three times.

### 4.11. Assessment of vB_EfaS-271 Phage Lytic Activity in Biofilms

The biofilm of *E. faecalis* 271 was prepared according to a method described earlier [82,83], with some modifications. Briefly, the overnight culture of host bacteria was diluted with TSB medium supplemented with 0.5% glucose to an OD_600_ of 0.2–0.3. Then, 2 mL of bacterial suspension was transferred to the each well of the 12-well polystyrene microtitre plate. *E. faecalis* 271 static biofilms were grown at 37 °C for 24 h. After incubation, the supernatant was discarded, and the biofilm was washed three times with 1 mL phosphate-buffered saline (PBS) to remove planktonic cells. In the next step, the lysate of phage vB_EfaS-271 was added to each well, except the control variant, to a final titer of 10^2^, 10^4^, 10^6^ or 10^8^ PFU per well and incubated for 24 h at 37 °C by. Next, phage lysate was removed and biofilm was washed three times with 1 mL of PBS and finally suspended in 1ml of PBS. The phage lytic activity in the bacterial biofilms was tested by measuring the optical density with a plate reader (EnSpire multimode plate reader) at a wavelength of 600 nm. In order to acquire photographs of the enterococcal biofilm for densitometric analyses, the phage lysate was removed from each well, washed three times with 1 mL of PBS and surface-attached cells were dried at 37 °C for 15 min. Densitometry analysis of biofilm density after phage infection was performed by using QuantityOne software (Bio-Rad, Hercules, CA, USA). The average results were obtained from three independent experiments.

### 4.12. Crystal Violet (CV) Staining for the Quantification of Biofilm Biomass

Plates with enterococcal biofilms *were* prepared as described in Section 4.11. After incubation at 37 °C for 24 h, the bacteria and phage suspension were discarded, and the wells were washed three times with 1 mL of PBS. Then, the wells were air-dried at 37 °C for 15 min and stained with 0.1% crystal violet (CV; Sigma-Aldrich) for 30 min at room temperature. Following incubation, the bacterial biofilm was rinsed five times with 1 mL of PBS to remove excess stain. CV incorporated into the biofilms was dissolved by the addition of 1 mL of 96% ethanol to each well. The OD_575_ was measured by using an EnSpire (PerkinElmer, Waltham, MA, USA) multimode plate reader. The biofilm area after fixation was also photographed to indicate the differences between the control sample and biofilm biomass treated with phage lysate. This experiment was repeated three times.

### 4.13. Detection of the Metabolic Activity of Biofilm Cells after vB_EfaS-271 Infection

Biofilm cells were prepared and incubated with vB_EfaS-271 lysate as described in Section 4.11. The resazurin assay was conducted according to the protocol described elsewhere [82]. First, the liquid medium was removed from each well. Next, the surface-attached cells were suspended in 1 mL of PBS. Then, resazurin solution (Sigma-Aldrich) was added to each well to a final concentration of 24 μg/mL. The plates were gently shaken and incubated in an EnSpire multimode plate reader for 50 min at room temperature. The fluorescence of the produced resorufin (λexc = 570 nm and λem = 590) was measured every 5 min. The results are presented as fluorescent units (FU). The procedure was repeated three times.

### 4.14. Extraction, Sequencing and Bioinformatic Analysis of the vB_EfaS-271 Genome

DNA of vB_EfaS-271 was isolated from purified phage lysate according to a procedure described earlier [80,82]. The sample was treated with DNase I (1 U/µL; Thermo Fisher Scientific, Waltham, MA, USA) and RNaseA (5 µg/mL; Thermo Fisher Scientific) for 30 min at 37 °C to destroy bacterial nucleic acids. Then, both enzymes were inactivated at 95 °C, and the genomic DNA of vB_EfaS-271 was isolated with a MasterPure^TM^ Complete DNA and RNA purification kit (Epicenter, Gdansk, Poland). The concentration of phage DNA was examined spectrophotometrically by measuring the absorbance at a wavelength of 260 nm.

vB_EfaS-271 genomic DNA was sequenced by the company Genomed, involving next generation sequencing (NGS) and the MiSeq (Illumina, San Diego, CA, USA) genome sequencer. The quality of the reads was determined using FastQC (Babraham Bioinformatics. Available online: https://www.bioinformatics.babraham.ac.uk/projects/fastqc/ (last accessed on 27 August 2020)). To remove the adapters, N bases, and low-quality reads, the raw data (290,884 raw reads) were filtered using the Cutadapt program (http://code.google.com/p/cutadapt/) with the following parameters: −q = 20 and −m =3 6. *De novo* assembly with 289,360 trimmed reads (99.48% of raw reads) was performed using CLC Genomics Workbench. Finally, the assembly generated a single contig, corresponding to the entire phage vB_EfaS-271 genome with an average coverage of 1527×.

Open reading frames (ORFs) were predicted with myRAST [84] and UGENE [85] bioinformatic software (Unipro UGENE v. 35. Available online: http://ugene.net/ (last accessed 27 August 2020)). Putative functions of translated protein products were annotated using the BLASTP and PHASTER prophage/virus databases [86]. The NCBI conserved domain database was used for functional classification of predicted phage proteins via subfamily domain architectures [87]. ShortBRED was used to search the virulence factors and toxins in predicted ORFs against Virulence Factors of the Pathogenic Bacteria database (VFDB) [88,89]. The circular map of the vB_EfaS-271 genome was created by the BLAST Ring Image Generator (BRIG) platform (SOURCEFORGE. Available online: https://sourceforge.net/projects/brig/ (last accessed 17 August 2020)). GC content analyses were prepared with CGView [90]. Moreover, transfer RNAs (tRNAs) were scanned by using the tRNAscan_SE web server (LOWELAB. Available on line: http://lowelab.ucsc.edu/tRNAscan-SE/ (accessed 15 July 2020)) [91,92]. The PhagePromoter tool (Galaxy Docker Build. Available online: https://bit.ly/2Dfebfv (last accessed 27 August 2020)) was used to determine phage promoters with different motifs [93]. Rho-independent terminators were predicted by the ARNold web server (Research group on RNA Sequence, Structure and Function. Available on line: http://rssf.i2bc.paris-saclay.fr/toolbox/arnold/ (last accessed 27 August 2020)) [94,95,96,97].

Comparison of the ORFs from four related phages, vB_EfaS_LM99 (MH355583.1) [41], vB_EfaS_AL3 (NC_042126.1) [42], LY0322 (NC_042125.1) and phiSHEF5 (NC_042023.1) [43] was performed by using the EasyFig program (Easyfig. Available online: http://mjsull.github.io/Easyfig/files.html (last accessed 27 August 2020). Finally, the DNA sequence of the. *E. faecalis* 271 phage vB_EfaS-271 was deposited in GenBank under accession number MT520979.1.

### 4.15. Phylogenetic Analysis

To estimate the phylogenetic position of the new *Enterococcus* phage vB_EfaS-271, the nucleotide sequences of the genes coding for terminase large subunit TerL were compared with the sequences of other reference *Enterococcus* phages that were deposited in the NCBI database. We have also compared the nucleotide sequences of lysin gene of vB_EfaS-271 with the sequences of the equivalent genes of other phages. Two data matrices for the analysis of lysin gene were created. One matrix contained the sequences of the gene fragment (about 630 base pairs), including the sequence coding for an N-terminal catalytic domain (ECD). The second matrix contained the sequences of the complete lysin gene. For the multiple genes phylogenetic analysis we selected genes coding for proteins of known functions such as: HNH homing endonuclease, terminase small subunit, terminase large subunit, portal protein, prohead protease, major capsid protein, major tail protein, head-tail connector protein, head-tail adaptor protein, head-tail joining protein, tail tape measure chaperone protein, tail tape measure protein, tail protein, minor tail protein, tail fiber protein, holing, lysin, glutaredoxin, DNA polymerase B-like protein, bifunctional DNA primase/polymerase, helicase, endonuclease and DNA primase. All genes are listed in the Supplementary Table S5. DNA sequences were translated to amino-acid and then aligned and adjusted by eye using Seaview [98]. The jModelTest v. 2.1.1 [99] was used in order to choose the best-fitting evolutionary model by the Akaike Information Criterion. For the matrices of terminase large subunit and the lysin gene fragment the evolutionary model GTR + I + G was used. For the matrix of the complete lysin gene the GTR + G model was used. In case of multiple genes analysis p-distance was used based on a recommendation of [100]. All matrices were analyzed using PAUP * (Phylogenetic Analysis Using Parsimony * and Other Methods) version 4.0a [101]. The optimality criterion was set to distance using the Neighbour-Joining algorithm (NJ). The robustness of the tree topology was assessed by bootstrap analyses based on 1000 replicates. The prediction of the lysin domains was done with the Pfam database [61] (European Molecular Biology Laboratory, ELIXIR, Pram 33.1. Available online: http://pfam.xfam.org/ (accessed on 26 August 2020).

### 4.16. Mass Spectrometry Analysis of vB_EfaS-271 Proteins

Analysis of the vB_EfaS-271 proteins was carried out in the Institute of Bioorganic Chemistry of the Polish Academy of Sciences. Briefly, *E. faecalis* 271 host was grown in TSB medium at 37 °C to an OD_600_ of 0.3. Then, phage stock solution was added to 100 mL of the bacterial culture at an M.O.I. of 0.1. After 1.5 h incubation of the sample at 37 °C with shaking, the lysis of the host cells was observed. At this time, the obtained phage lysate was treated with 4 mL of chloroform for 15 min. In the next step, the mixture was centrifuged at 4000× *g* for 10 min at 4 °C and bacterial debris of *E. faecalis* 271 was removed. The lysate of vB_EfaS-271 was concentrated for 22 h at 4 °C by using 10% polyethylene glycol 8000 (PEG8000). Fallowing the overnight incubation, the phage particles were pelleted by centrifugation at 8000× *g* for 20 min at 4 °C and suspended in 1.2 mL of TM buffer (10 mM Tris-HCl, 10 mM MgSO_4_; pH 7.2). Then, the vB_EfaS-271 virions were extracted with 50% chloroform and purified by using the cesium chloride density gradient centrifugation method [79]. In the next step, the obtained phage lysate was incubated with 4 volumes of ice-cold acetone at −20 °C for 30 min. Then, the sample was centrifuged at 13,000× *g* for 5 min at 4 °C, and the supernatant was discarded. The obtained pellet was suspended in 50 mM ammonium bicarbonate, and the concentration of viral proteins was estimated by using a BCA colorimetric assay kit (Thermo Fisher Scientific). In the next step, the phage protein lysate was treated with 5.6 mM dithiothreitol (DTT) in 50 mM ammonium bicarbonate. Following a 5-min incubation at 95 °C, the sample was cooled to room temperature and then alkylated with 5 mM iodoacetamide for 20 min in the dark. The phage proteins were digested overnight with 0.2 µg of sequencing-grade trypsin at 37 °C. The enzyme was inactivated by the addition of trifluoroacetic acid (TFA), and then the mixture was loaded into an HPLC conical vial. Analysis of the vB_EfaS-271 proteins was performed by employing the Dionex UltiMate 3000 RSLC nanoLC System connected to a Q Exactive Orbitrap mass spectrometer (Thermo Fisher Scientific). The peptides obtained after trypsin digestion were separated on a reversed-phase Acclaim PepMap RSLC nanoViper C18 column using an acetonitrile gradient. Mass spectra were acquired on the Q Exactive instrument in data-dependent mode by using the top 10 data-dependent MS/MS scans. The target value for the full scan MS spectra was set to 1e6 with a maximum injection time of 100 ms and a resolution of 70,000 at *m*/*z* 400. The 10 most intense ions charged by two or more were selected with an isolation window of 2 Da and fragmented by higher energy collisional dissociation with an NCE 27. The ion target value for MS/MS was set to 5 × 10^4^ with a maximum injection time of 100 ms and a resolution of 17,500 at *m*/*z* 400. The identification procedure of vB_EfaS-271 proteins was conducted by Proteome Discoverer 1.4 software (Thermo Fisher Scientific). The database of amino acid sequences of phage vB_EfaS-271 proteins was prepared manually. The proteins were classified as positively identified if at least two peptide spectral matches per protein were found by the Sequest search engine, and a peptide score reached the significance threshold FDR = 0.05.

### 4.17. Statistical Analysis

The statistical analysis of significance was undertaken by using the *t*-test. The significance of differences between compared experimental samples are marked by asterisks as follows: *p* < 0.05 (*), *p* < 0.001 (**) or *p* < 0.001 (***).

## Figures and Tables

**Table 1:** Lytic activity of vB_EfaS-271 against tested *E. faecalis* strains.

Bacterial Strain	Phage Sensitivity ^a^
*Enterococcus faecalis* 230	−
*Enterococcus faecalis* 271	+
*Enterococcus faecalis* 272	+
*Enterococcus faecalis* 273	−
*Enterococcus faecalis* 274	−
*Enterococcus faecalis* 275	−
*Enterococcus faecalis* M2056	−
*Enterococcus faecalis* OG1RF	−
*Enterococcus faecalis* V583	+

^a^ Symbols: (+) clear zones or (−) no plaques after infection of tested bacteria with vB_EfaS-271 bacteriophage.

**Table 2:** Mass spectrometry analysis of vB_EfaS-271 proteins.

Detected Protein	Predicted Function	Molecular Mass (kDa)	Number of Peptides	Sequence Coverage (%)	Protein Score
vB_EfaS-271_08	portal protein	43.0	18	83.03	144.10
vB_EfaS-271_09	prohead protease	21.1	3	63.64	10.64
vB_EfaS-271_10	major capsid protein	45.3	38	91.34	1412.60
vB_EfaS-271_11	major tail protein	7.0	5	93.94	79.72
vB_EfaS-271_14	head-tail joining protein	15.3	2	54.81	1.94
vB_EfaS-271_15	head-tail joining protein	14.2	3	44.63	8.62
vB_EfaS-271_16	major tail protein	19.9	7	65.96	4.38
vB_EfaS-271_18	tail tape measure protein	156.0	44	78.64	131.99
vB_EfaS-271_19	tail protein	78.9	34	77.13	168.36
vB_EfaS-271_20	minor tail protein	88.4	44	88.65	161.67
vB_EfaS-271_21	tail fiber protein	9.2	2	97.53	1.61
vB_EfaS-271_23	lysin	39.9	8	45.21	4.04
vB_EfaS-271_25	DNA polymerase B-like protein	87.5	11	73.00	11.70
vB_EfaS-271_28	unknown protein	26.5	8	70.21	13.99
vB_EfaS-271_49	DNA primase	60.7	31	89.58	149.50

**Table 3:** Sensitivity of vB_EfaS-271 virions to external physical and chemical agents.

External Factors (Time and Conditions of Incubation) ^a^	Percentage of Viability of vB_EfaS-271 under Certain Conditions ± SD
−80 °C (16 h)	100.0 ± 0.0
−20 °C (16 h)	100.0 ± 1.9
20 °C (24 h)	100.0 ± 0.0
30 °C (24 h)	100.0 ± 0.5
37 °C (24 h)	100.0 ± 0.0
40 °C (40 min)	100.0 ± 7.9
42 °C (40 min)	42.6 ± 11.3
62 °C (40 min)	1.4 ± 0.5
95 °C (5 min)	0.0 ± 0.0
pH 2 (1 h; 37 °C)	0.0 ± 0.0
pH 4 (1 h; 37 °C)	71.4 ± 2.8
pH 10 (1 h; 37 °C)	100.0 ± 0.0
pH 12 (1 h; 37 °C)	90.0 ± 12.3
Osmotic shock (15 min; RT)	78.8 ± 10.5
0.09% SDS (20 min; 45 °C)	10.3 ± 2.6
0.1% CTAB (1 min; RT)	0.0 ± 0.0
0.1% Sarkosyl (10 min; RT)	100 ± 9.8
63% Ethanol (1 h; RT)	0.2 ± 0.0
90% Acetone (1 h; RT)	0.9 ± 0.4
50% DMSO (1 h; RT)	82.4 ± 12.5
Chloroform (1.5 h; 4 °C)	55.6 ± 9.6
10% dish soap (5 min; RT)	93.0 ± 6.4
10% soap (2 min; RT)	77.8 ± 11.1
Line-Antibacterial 70 (5 min; RT)	81.5 ± 6.4
0.5% Virkon (30 min; RT)	0.0 ± 0.0
5% Viruton Pulver (30 min; 30 °C)	0.0 ± 0.0

^a^ Abbreviation: RT, room temperature.

## Supplementary Materials

Supplementary Materials can be found at https://www.mdpi.com/1422-0067/21/17/6345/s1. Table S1: Bacterial strains; Table S2: vB_EfaS-271 lytic activity; Table S3: vB_EfaS-271 annotations; Table S4: Predicted promoters and Rho-independent terminators of vB_EfaS-271 bacteriophage; Table S5: List of genes used in phylogenetic analysis; Table S6: Mass spectrometry data of vB_EfaS-271 proteins.

## Abbreviations

BCA	Bicinchonic acid
BRIG	BLAST ring image generator
CFU	Cetyltrimethylammonium bromide
CTAB	Crystal violet
CV	*Dimethyl sulfoxide*
*DNase I*	Deoxyribonuclease
DTT	Dithiothreitol
EHEC	Enterohemorrhagic *Escherichia coli*
EM	Emmision
EPEC	Enteropathogenic *Escherichia coli*
ESP	*Eschrichia coli* secretion protein
EXC	Excitation
FDR	False discovery rate
FU	Fluorescence units
HPLC	High-performance liquid chromatography
LA	Luria-Bertani agar
LB	Luria-Bertani broth
LIN	Linezolid
MS	Mass spectrometry
M.O.I.	Multiplicity of infection
NCBI	National center for biotechnology information
NGS	Next generation sequencing
OD	Optical density
ORF	Open reading frame
PBS	Phosphate-buffered saline
PFU	Plaque forming units
RNaseA	Ribonuclease A
SD	Standard deviation
Stx	Shiga toxin
TEM	Transmission electron microscopy
TFA	Trifluoroacetic acid
TerL	Large subunit of terminase
TM	Tris-Magnesium sulfate buffer
TSA	Tryptic soy agar
TSB	Tryptic soy broth
VFDB	Virulence Factors of the Pathogenic Bacteria database
