# Supplementary material for: Characterization of the Bacteriophage vB_EfaS-271 Infecting Enterococcus faecalis
Source: Int J Mol Sci. 2020 Sep 1;21(17):6345. doi: 10.3390/ijms21176345 (PMC7503890; doi:10.3390/ijms21176345)
Supplement: Supplementary file 1 [file ijms-21-06345-s001.zip › Supplementary Tables S1-S2-S5.pdf]

Supplementary Material

Characterization of the bacteriophage vB_EfaS-271 infecting *Enterococcus faecalis*

Gracja Topka-Bielecka [1], Sylwia Bloch [2], Bożena Nejman-Faleńczyk [1], Michał Grabski [1, 3], Agata Jurczak-Kurek [4], Marcin Górniak [4],Aleksandra Dydecka [1], Agnieszka Necel [1], Grzegorz Węgrzyn [1] and Alicja Węgrzyn [2,*]

[1] Department of Molecular Biology, University of Gdansk, Wita Stwosza 59, 80-308 Gdansk, Poland; gracja.topka@phdstud.ug.edu.pl; bozena.nejman-falenczyk@ug.edu.pl; michal.grabski@phdstud.ug.edu.pl; aleksandra.dydecka@phdstud.ug.edu.pl; agnieszka.necel@phdstud.ug.edu.pl; grzegorz.wegrzyn@biol.ug.edu.pl

[2] Laboratory of Molecular Biology, Institute of Biochemistry and Biophysics, Polish Academy of Sciences, Kładki 24, 80-822 Gdansk, Poland; sylwia.bloch@ug.edu.pl, alicja.wegrzyn@biol.ug.edu.pl

[3] Laboratory of Marine Biogeochemistry, Institute of Oceanology, Polish Academy of Sciences, Powstańców Warszawy 55, 81-712 Sopot, Poland; michal.grabski@phdstud.ug.edu.pl

[4] Department of Molecular Evolution, University of Gdansk, Wita Stwosza 59, 80-308 Gdansk, Poland; agata.jurczak-kurek@ug.edu.pl; marcin.gorniak@ug.edu.pl

* Correspondence: alicja.wegrzyn@biol.ug.edu.pl; Tel.: +48 58 523 6040

Supplementary Table S1

Table S1. Bacterial strains used in this study.

Bacterial strains	Source or references	Relevant genotype or other characteristics
Enterococcus faecalis strains		
**230**	[66,67]	urban sewage isolate
**271**		PEN^{S}, AMP^{S}, VAN^{S}, NFV^{S}; GEN^{R}, SXT^{R}
**272**		PEN^{S}, AMP^{S}, VAN^{R}, NFV^{S}; SXT^{R}; GEN^{R}
**273**	Specialist Hospital of St. Wojciech in Gdansk (Poland)	PEN^{S} AMP^{S}, VAN^{S}, NFV^{S}; SXT^{R}; GEN^{R}
**274**		PEN^{S}, AMP^{S}, GEN^{S}, VAN^{S}, NFV^{S}; SXT^{SS}
**275**		PEN^{S}, AMP^{S}, GEN^{S}, VAN^{S}, NFV^{S}; SXT^{SS}
**M2056**	[65]	GEN^{S},VAN^{S}; SXT^{SS}; PEN^{R}, AMP^{R}, NFV^{R}
**OG1RF**	[68,69]	RIF^{R}, fusidic acid^{R}
**V583**	[70,71]	VAN^{R}
***Escherichia coli* strains**		
**MG1655**	[72]	F- *λ– ilvG rfb-50 rph-1*
**C600**	[73]	F- *tonA21 thi-1 thr-1 leuB6 lacY1 glnV44 rfbC1 fhuA1 λ –*
**TAP90**	[74]	-
**Hfr3000**	[75]	*rfbC1 fhuA1 λ –*
**EPEC-A 129**	Specialist Hospital of St. Wojciech in Gdansk (Poland)	stool; EspA
**EPEC-B 21950**	Specialist Hospital of St. Wojciech in Gdansk (Poland)	stool; EspB
**EPEC-C 22032**	Specialist Hospital of St. Wojciech in Gdansk (Poland)	stool; EspC
**O157:H7(ST2–8624)**	[76]	stool; Stx1 & Stx2
**Others strains**		
***Enterococcus faecium* 270**	Specialist Hospital of St. Wojciech in Gdansk (Poland)	GEN^{S}, VAN^{S}, LIN^{S}, NFV^{S}, PEN^{R}, AMP^{R}, SXT^{R}
***Shigella flexneri* 12022**	ATCC® 12022™	-
***Staphylococcus sciuri* IO**	Institute of Oceanology of Polish Academy of Sciences in Sopot (Poland)	urban sewage isolate

***Pseudomonas aeruginosa* 575**	National Medicines Institute in Warsaw (Poland)	decubitus ulcers
***Pseudomonas aeruginosa* 1947**	National Medicines Institute in Warsaw (Poland)	wound
***Salmonella enterica* Anatum**	National Salmonella Centre at Medical University of Gdansk (Poland)	-
***Salmonella enterica* Heidelberg**	National Salmonella Centre at Medical University of Gdansk (Poland)	-
***Salmonella enterica* Panama**	National Salmonella Centre at Medical University of Gdansk (Poland)	-
***Salmonella enterica* Reading**	National Salmonella Centre at Medical University of Gdansk (Poland)	-

Abbreviations: EHEC, Enterohemorrhagic *E. coli*; EPEC, Enteropathogenic *E. coli*; Stx, Shiga toxin; Esp, *E. coli* secretion protein; S (sensitive), SS (semisensitive), R (resistant); GEN, gentamicin; VAN, vancomycin; LIN, linezolid; NFV, nelfinavir; AMP, ampicillin; SXT, trimethoprim-sulfamethoxazole; PEN, penicillin

Supplementary Table S2

Table S2. Lytic activity of vB_EfaS-271 against other Gram-negative and Gram-positive bacteria.

Bacterial strain	Plaques
Enterococcus faecium 270	-
Escherichia coli MG1655	-
Escherichia coli C600	-
Escherichia coli TAP90	-
Escherichia coli Hfr3000	-
Escherichia coli O157:H7 (ST2-8624)	-
Escherichia coli EPEC-A	-
Escherichia coli EPEC-B	-
Escherichia coli EPEC-C	-
Shigella flexineri 12022	-
Salmonella enterica (Anatum)	-
Salmonella enterica (Heidelberg)	-
Salmonella enterica (Reading)	-
Salmonella enterica (Panama)	-
Staphylococcus sciuri IO	-
Pseudomonas aeruginosa 575	-
Pseudomonas aeruginosa 1947	-

Symbol (-) no plaques after infection with vB_EfaS-271 bacteriophage.

Supplementary Table S5

Table S5. List of genes with their positions in phage genomes used for multi-gene phylogenetic analysis

Function of gene in vB_Efa-271	Gene position in LY0322		Gene position in vB_EfaS_AL3		Gene position in vB_EfaS_LM99		Gene position in phiSHEF4		Gene position in phiSHEF5		Gene position in EFRM31	
	**Start**	**End**	**Start**	**End**	**Start**	**End**	**Start**	**End**	**Start**	**End**	**Start**	**End**
HNH homing endonuclease	232	603	40302	40673	38390	38761	33394	33765	105	476	80	451
terminase small subunit	39436	39909	1006	1479	39271	39744	34326	34799	40694	41167	8788	9261
terminase large subunit	37294	39015	1881	3602	27	1751	35396	37120	38600	40321	9556	11280
portal protein	35904	37055	3841	4992	2359	3510	37721	38872	36848	37999	11519	12670
prohead protease	35354	35941	4955	5542	3497	4060	38859	39422	36298	36861	12633	13220
major capsid protein	34037	35284	5612	6859	4130	5383	39492	40745	34975	36228	13290	14537
major tail protein	33711	33911	6985	7185	5509	5709	40870	41070	34631	34852	14663	14863
head-tail connector protein	33371	33673	7229	7525	5753	6049	160	456	34321	34617	14901	15203
head-tail adaptor protein	33064	33399	7497	7832	6021	6356	428	763	34014	34349	15175	15510
head-tail joining protein	32660	33067	7829	8236	6353	6760	760	1167	33610	34017	15507	15914
head-tail joining protein	32298	32663	8233	8598	6757	7122	1164	1529	33248	33613	15911	16276
major tail protein	31653	32219	8677	9243	7198	7764	1608	2168	32606	33172	16355	16921
tail tape measure chaperone protein	31162	31473	9423	9734	7959	8270	2346	2657	32100	32411	-	-
tail tape measure protein	26535	30905	9991	14361	8527	12897	2914	7284	27473	31843	-	-
tail protein	24379	26460	14436	16517	12977	15052	7367	9442	25318	27393	-	-
minor tail protein	22013	24367	16529	18883	15064	17418	9448	11715	22952	25306	-	-
tail fiber protein	21589	21834	19063	19308	17597	17842	11898	12143	22529	22774	-	-
holin	21341	21574	19323	19556	17857	18093	12157	12390	22278	22514	-	-
lysin	20343	21338	19559	20656	18090	19187	12393	13490	21295	22281	-	-
glutaredoxin	19499	19768	20745	20972	19277	19504	13583	13807	20984	21211	-	-
DNA polymerase B-like protein	17144	19435	21379	23670	19567	21858	14557	16848	18630	20921	-	-
HNH homing endonuclease	12624	13103	28001	28480	25956	26435	20783	21262	14823	15302	-	-
bifunctional DNA primase/polymerase	10814	11557	30170	30910	27509	28252	22336	23079	12788	13663	-	-
helicase	8561	9859	31558	32856	28730	30025	23511	24806	10846	12141	-	-
endonuclease	7941	8315	33012	33398	30018	30404	24799	25188	10464	10853	7920	8294
DNA primase	4629	6215	35112	36698	32515	34095	27403	28983	6326	7906	4343	5929

References (the numeration of the references is compatible with the numeration of the references presented in the main text of the publication)

65. Nowicki, D.; Maciąg-Dorszyńska, M.; Bogucka, K.; Szalewska-Pałasz, A.; Herman-Antosiewicz, A. Various modes of action of dietary phytochemicals, sulforaphane and phenethyl isothiocyanate, on pathogenic bacteria. *Sci Rep*. **2019**, *9 (1)*, 13677. doi: 10.1038/s41598-019-50216-x
66. Luczkiewicz, A.; Felis, E.; Ziembinska, A.; Gnida, A.; Kotlarska, E.; Olanczuk-Neyman, K.; Surmacz-Górska, J. Resistance of *Escherichia coli* and *Enterococcus* spp. to selected antimicrobial agents present in municipal wastewater. *J Water Health*. **2013**, *11 (4)*, 600–612. doi: 10.2166/wh.2013.130
67. Sadowy, E.; Luczkiewicz, A. Drug-resistant and hospital-associated *Enterococcus faecium* from wastewater, riverine estuary and anthropogenically impacted marine catchment basin. *BMC Microbiol*. **2014**, *14*, 66. doi: 10.1186/1471-2180-14-66
68. Murray, B.E.; Singh, K.V.; Ross, R.P.; Heath, J.D.; Dunny, G.M.; Weinstock, G.M. Generation of restriction map of *Enterococcus faecalis* OG1 and investigation of growth requirements and regions encoding biosynthetic function. *J Bacteriol*. **1993**, *175 (16)*, 5216–5223. doi: 10.1128/jb.175.16.5216-5223.1993
69. Gawryszewska, I.; Żabicka, D.; Hryniewicz, W.; Sadowy, E. Penicillin-resistant, ampicillin-susceptible *Enterococcus faecalis* in Polish Hospitals. *Microb Drug Resist*. **2020** (online ahead of print). doi: 10.1089/mdr.2019.0504
70. Kawalec, M.; Gniadkowski, M.; Zielińska, U.; Kłos, W.; Hryniewicz W. Vancomycin-resistant *Enterococcus faecium* strain carrying the *vanB2* gene variant in a Polish hospital. *J Clin Microbiol*. **2001**, *39 (2)*, 811–815. doi: 10.1128/JCM.39.2.811-815.2001
71. Sahm, D.F.; Kissinger, J.; Gilmore, M.S.; Murray, P.R.; Mulder, R.; Solliday, J.; Clarke, B. *In vitro* susceptibility studies of vancomycin-resistant *Enterococcus faecalis*. *Antimicrob Agents Chemother*. **1989**, *33*, 1588–1591. doi: 10.1128/aac.33.9.1588
72. Jensen, K.F. The *Escherichia coli* K-12 wild types W3110 and MG1655 have an *rph* frameshift mutation that leads to pyrimidine starvation due to low *pyrE* expression levels. *J. Bact*. **1993**, *175 (11)*, 3401–3407. doi: 10.1128/jb.175.11.3401-3407.1993
73. Appleyard, R.K. Segregation of new lysogenic types during growth of a doubly lysogenic strain derived from *Escherichia coli* K12. *Genetics* **1954**, *39(4)*, 440–452.
74. Patterson, T.A.; Dean, M. Preparation of high titer lambda phage lysates. *Nucleic Acids Res*. **1987**, *15 (15)*, 6298. doi: 10.1093/nar/15.15.6298
75. Bachmann, B.J. Pedigrees of some mutant strains of *Escherichia coli* K-12. *Bacteriol Rev*. **1972**, *36 (4)*, 525–557.
76. Griffin, P.M.; Ostroff, S.M.; Tauxe, R.V.; Greene, K.D.; Wells, J.G.; Lewis, J.H.; Blake, P.A. Illnesses associated with *Escherichia coli* O157:H7 infections. A broad clinical spectrum. *Ann Intern Med*. **1988**, *109 (9)*, 705–712. doi: 10.7326/0003-4819-109-9-705
